# Flow-Induced New Channels of Energy Exchange in Multi-Scale Plasma Dynamics – Revisiting Perturbative Hybrid Kinetic-MHD Theory

**DOI:** 10.1038/srep25644

**Published:** 2016-05-10

**Authors:** Junya Shiraishi, Naoaki Miyato, Go Matsunaga

**Affiliations:** 1 Japan Atomic Energy Agency, 801-1 Mukoyama, Naka, Ibaraki 311-0193, Japan; 2 Japan Atomic Energy Agency, 2-166 Omotedate, Rokkasho, Aomori 039-3212, Japan

## Abstract

It is found that new channels of energy exchange between macro- and microscopic dynamics exist in plasmas. They are induced by macroscopic plasma flow. This finding is based on the kinetic-magnetohydrodynamic (MHD) theory, which analyses interaction between macroscopic (MHD-scale) motion and microscopic (particle-scale) dynamics. The kinetic-MHD theory is extended to include effects of macroscopic plasma flow self-consistently. The extension is realised by generalising an energy exchange term due to wave-particle resonance, denoted by δ W_K_. The first extension is generalisation of the particle’s Lagrangian, and the second one stems from modification to the particle distribution function due to flow. These extensions lead to a generalised expression of δ W_K_, which affects the MHD stability of plasmas.

A plasma, a collection of charged particles, can be characterised by its multi-scale nature. This multi-scaleness plays an important role in plasma dynamics. The small-scale dynamics relevant to constituent particles’ motion can strongly affect the macroscopic (fluid) dynamics. Each particle moves in a complicated manner under the influence of electromagnetic field and underlines complex macroscopic behaviour. The macroscopic motion changes the electromagnetic field and affects the particle dynamics vice versa. This interaction is fully nonlinear and is significantly involved. To analyse this complicated interaction, a framework called the hybrid kinetic-magnetohydrodynamic (MHD) theory has been developed^1^. Especially, the so-called “perturbative” approach enables the analysis tractable, which neglects the effect of particle kinetics on modifying the structure of MHD eigenmode and the effect of three dimensional distortion on the particle trajectory. One of the most celebrated examples of the application of this approach is the energetic particles’ stabilisation of internal kink^1^,^2^. The internal kink is a macroscopic MHD instability and is strongly affected by energetic particle motion via wave-particle resonance.

The kinetic-MHD theory basically consists of macroscopic single-fluid MHD equations, and the microscopic (kinetic) effect is consolidated in the total pressure tensor P defined by


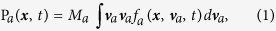


where the subscript 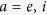 indicates the species of electrons and ions, *M* is the particle mass, and *f* is the particle distribution function. Note that 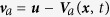 is a “random” motion from macroscopic flow 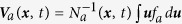 where ***u*** is the particle velocity (in the laboratory frame) and 
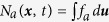
 is the number density. Evolution of *f* can be analysed by the drift-kinetic theory, which describes the dynamics of gyration centre (called guiding centre). In a magnetised plasma, charged particles rapidly gyrate around the magnetic field lines, and this fast motion can be separated by the ordering. The remaining guiding centre motion conserves the magnetic moment 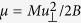 where the subscript ⊥ indicates the perpendicular component to the equilibrium magnetic field ***B*** and *B* = |***B***|. The guiding centre motion has additional adiabatic invariants, which yields the periodic motion in the longitudinal direction (called bounce motion), in which some guiding centres are not passing but trapped by the mirror force of the non-uniform magnetic field. Also the guiding centres slowly move in the azimuthal direction (called the precession drift).

The kinetic-MHD theory has a long history, and it is recently attracting lots of attention in the community of fusion plasma physics, since it is expected to explain the experimental results that small flow (comparable to the particle drift motion) can stabilises the resistive wall mode (RWM), which is one of the most dangerous MHD instabilities in fusion plasmas^3^. This theory has revealed important physics for RWM stabilisation by flow such as collisional effects and bounce and precession resonance effects^4^,^5^. From the experimental side, in the JT-60 tokamak device, it was observed that change of flow shear at the *q* = 2 surface affects the stability of RWMs^6^. Here, the tokamak is a torus-shaped device for fusion plasma confinement by using the twisted magnetic field, and *q* is the safety factor that measures the pitch of the twist. These theoretical and experimental achievements encourage us to make more detailed investigation of the flow effect on RWM stability in the framework of the kinetic-MHD theory. Recently, the kinetic-MHD theory has been extensively studied. For example, it has been extended to include the energetic particle effects^7^,^8^, the resistive layer effect^9^, the plasma inertia effect^10^, the three-dimensional response^11^, and generalised to invoke the “self-consistent” approach, which includes the effect of particle kinetics on mode modification^12^. In this paper, we point out that these conventional theories have neglected the effect of macroscopic flow ***V***_*a*_ when computing P_*a*_, which means that the total pressure tensor reduces to the total stress tensor as 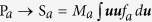 [see equation (1)].

The kinetic-MHD theory studies the RWM stability by the dispersion relation^3^,


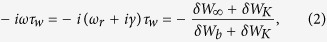


where 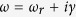 is the eigenvalue with a real frequency *ω*_*r*_ and a growth rate *γ* of RWM, *τ*_*w*_ is the diffusion time characterized by wall resistivity surrounding the plasma, and *δW*_∞_ and *δW*_*b*_ are the fluid potential energy with a wall located at infinity and *r* = *b* (*r* is a well-defined radial coordinate) respectively. Note that *δW*_∞_ and *δW*_*b*_ are real due to the self-adjointness of the fluid equations. In contrast, *δW*_*K*_ can be complex due to the kinetic resonance, hence it significantly affects the RWM stability. Therefore, an accurate estimate of *δW*_*K*_ is essential for the kinetic-MHD theory. The substance of *δW*_*K*_ is the quadratic form associated with the divergence of the perturbed total pressure tensor, 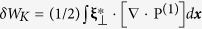, where **ξ** is the perturbed displacement obtained by fluid equations, the superscript (1) indicates the perturbed quantity, and the asterisk indicates the complex conjugate. Note that we have omitted the subscript *a*, which will be recovered when needed. From equation (1), we obtain 
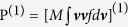
. The drift-kinetic theory^2^ indicates the perturbed particle distribution function can be schematically written as 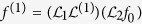 where 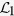 is an integral operator along the unperturbed particle orbit and 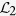 is a differential operator in the phase space including the derivative with respect to the radial coordinate, *L*^(1)^ is the perturbed guiding centre Lagrangian and *f*_0_ is a particle distribution function for the equilibrium state. Therefore, *L*^(1)^ and *f*_0_ are the fundamental constituents of the kinetic-MHD theory. We note that this structure of the formulation is not changed even if we invoke the dynamics in the moving frame. The drift-kinetic equation in the moving frame has the similar structure with the static case^2^, however, the independent variables in the phase-space, such as *v*_||_ and *μ* should be defined in the moving frame.

## Results

### Extension of the kinetic-MHD theory

First, we start from briefly seeing the mathematical structure of *δW*_*K*_ in the conventional kinetic-MHD theory. It is expressed as 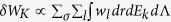, where *σ* is a sign of parallel component (to the equilibrium magnetic field) of particle velocity and *l* indicates the bounce harmonics, *E*_*k*_ is the kinetic energy, and Λ ∝ *μ*/*E*_*k*_ is the pitch angle variable. The integrand *w*_*l*_ in the conventional theory reads


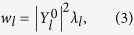


where 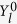 is related the perturbed guiding centre Lagrangian averaged over the bounce motion in the conventional form 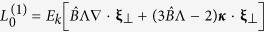, and *λ*_*l*_ is the resonance fraction, 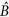 is the magnetic field amplitude normalized by its value at the magnetic axis, and ***κ*** is the magnetic curvature. In the conventional theory, an imaginary part of *δW*_*K*_ stems from the resonance fraction^14^,


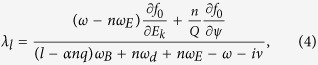


where *n* is the Fourier mode in the azimuthal direction for perturbations, *ω*_*E*_ the ***E*** × ***B*** frequency, ***Q*** the particle charge, *α* a parameter with = 1(0) for passing (trapped) guiding centres, *ω*_*B*_ the bounce frequency, *ω*_*d*_ the precession frequency, and ν the effective collision frequency. We emphasise that the guiding centre Lagrangian and particle distribution function, which are the fundamental constituents in *δW*_*K*_ formulation, assumes ***V*** = 0 in the conventional theories. Hence, the flow effect in equations (3) and (4) is commuted by the ***E*** × ***B*** frequency *ω*_*E*_.

Next, we move to the extension of the kinetic-MHD theory. The first extension stems from the generalisation of the guiding centre Lagrangian, which was performed in ref. 15. As was pointed out in the Introduction, ***v*** should be “random” motion from ***V***. Therefore, the guiding centre Lagrangian should be defined in a frame moving with ***V***. Such Lagrangian is studied in ref. 16, 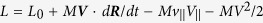 where *L*_0_ is the standard guiding centre Lagrangian, ***R*** is the position of the guiding centre and *v*_||_ is the parallel component of the particle velocity in the frame moving with ***V***. After some manipulation, we obtain^15^


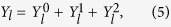


where 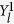 and 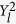 are related to the perturbed guiding centre Lagrangian associated with the flow, 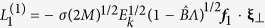 and 
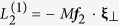
. Here, 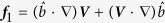 is the Coriolis acceleration (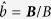) and 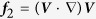 is the centrifugal acceleration.

The second extension originates from the generalisation of the particle distribution function, which reads^17^


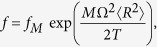


where *f*_*M*_ is the Maxwellian distribution function, Ω(*ψ*) is the angular frequency of the plasma flow in azimuthal direction and 〈·〉 indicates the average on the magnetic flux defined by *ψ* = const, where *ψ* is proportional to the magnetic flux in the longitudinal direction. Due to these two extensions, the resonance fraction is generalised as


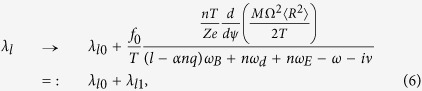


where *λ*_*l*0_ is formally same with equation (4) but the drift frequency *ω*_*d*_ should be modified due to the generalisation of Lagrangian, *Z* is the charge state and *e* is elementary charge. Note that this extension naturally includes the flow shear effect ∝*d*Ω/*dψ*. By these extensions, from equations (5) and (6), we obtain the generalised integrands of *δW*_*K*_ as


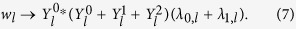


Therefore, as a final form of *δW*_*K*_, we obtain


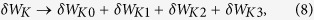


where we obtain the conventional but modified energy term 
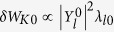
, the term associated with Coriolis acceleration 
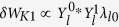
, the term related to the centrifugal acceleration 
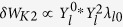
, and the term with flow shear effect (primary for ions) 
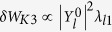
.

### Numerical analysis of flow and flow shear effects on stability

We have developed a module to compute *δW*_*K*_ by equations (3) and (7) in tokamak geometry. The integration by the pitch angle variable and the kinetic energy is implemented with the standard numerical technique^18^. This module is coupled with the MINERVA/RWMaC code^19^, which solves the linearised ideal MHD equations with equilibrium flow in tokamak geometry with vacuum and wall equations. We employ a simple formula for collision frequency^5^ as 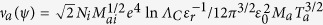 where *M*_*ai*_ = *M*_*a*_*M*_*i*_/(*M*_*a*_ + *M*_*i*_), Λ_*C*_ is the plasma parameter, *ε*_*r*_ is the inverse aspect ratio, and *ε*_0_ is the vacuum permittivity. The ***E*** × ***B*** frequency is computed by radial force balance of ions as 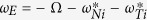. As for the singularities occurring on the *q* = integer surfaces, we have employed “regularized” eigenfunction at the surfaces as 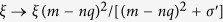 with a smoothing parameter *σ*′^20^.

**Figure 1:**
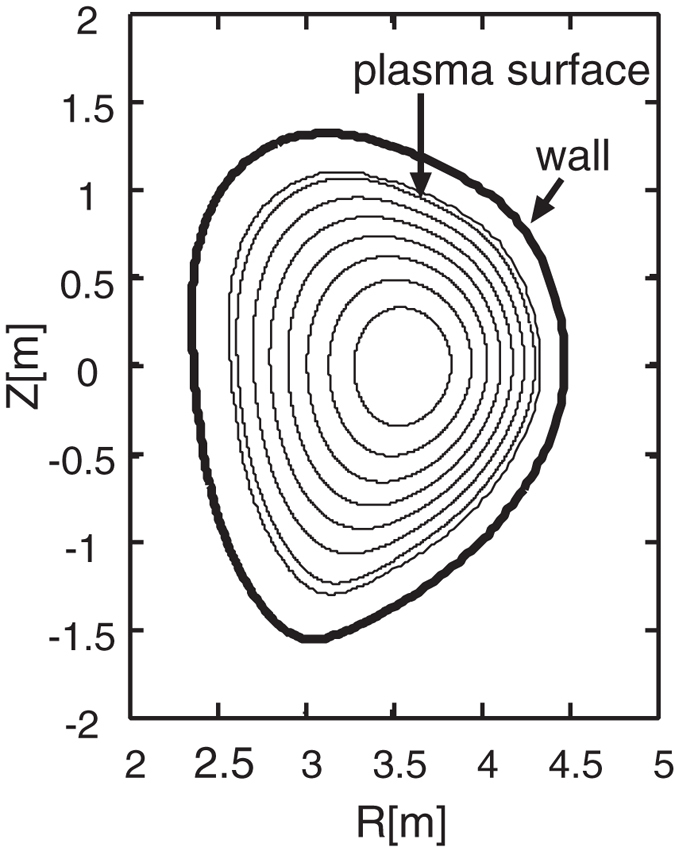
A contour of magnetic flux surfaces of numerical MHD equilibrium in the tokamak cross-section.

**Figure 2:**
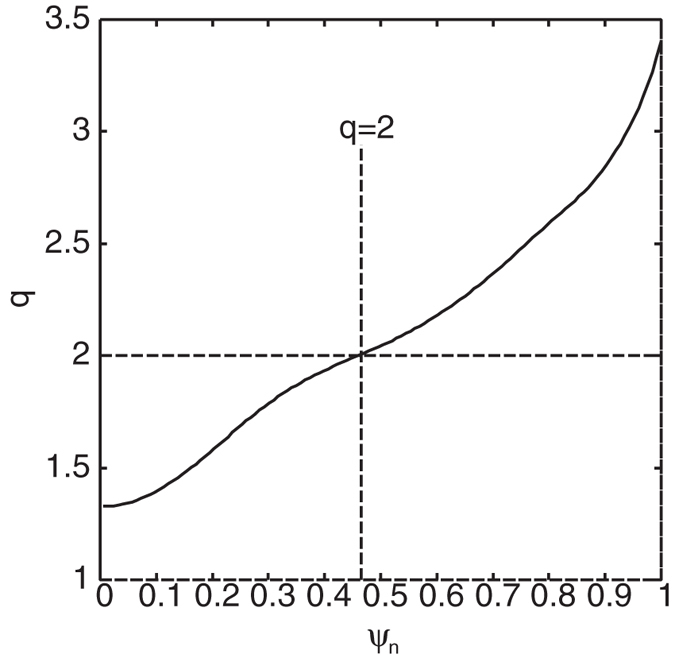
Safety factor profile of the numerical MHD equilibrium with indication of a rational surface *q* = 2 where *ψ*_*n*_ is the normalized magnetic flux.

In what follows, we investigate the theoretical aspect of the extended theory based on numerical MHD equilibrium. To this end, we pick up one discharge in the JT-60 experiment, which indicates the unstable RWMs appear. Magnetic flux surfaces and an approximated conformal wall *b* = 1.2 are shown in Fig. 1 and safety factor profile is shown in Fig. 2, which indicates the normal shear configuration. This equilibrium has *β*_*N*_ = 3.59 larger than the no-wall limit, hence the fluid RWM is unstable. Here, 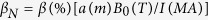 is the “normalized *β*″ where *β*(%) is the ratio of plasma pressure and magnetic pressure, *a*(m) the minor radius of the plasma, *B*_0_(T) the toroidal magnetic field, and *I*(MA) the plasma current. In this paper, we assume *τ*_*w*_ = 10 (ms) that is an approximate value for the JT-60 wall, and focus on *n* = 1 instability. When the wall is removed, this equilibrium has an unstable external kink with a growth rate *γτ*_*A*_ = 0.208 where *τ*_*A*_ is the Alfvén transit time defined at the magnetic axis. We note that to compute *δW*_*K*_, we have employed the eigenfunction of unstable external kink with *b* = ∞. As for the *δW*_*b*_, we extrapolate its value by computing the dependence of fluid *δW* on the wall location.

**Figure 3:**
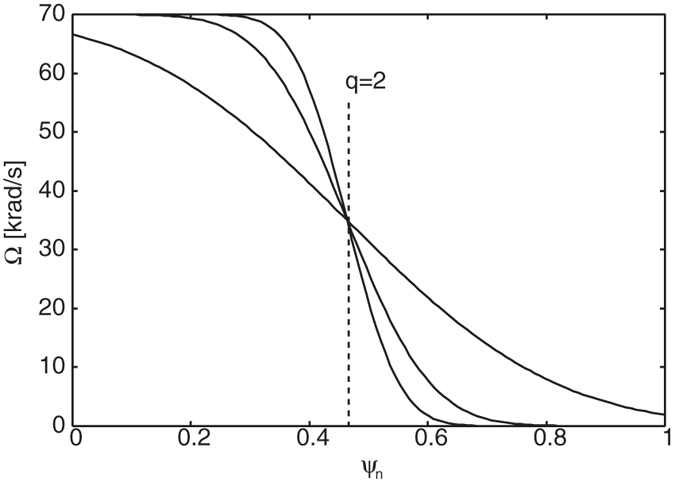
Rotation frequency profiles with various flow shear. The rotation frequency amplitude at the *q* = 2 surface is fixed.

Hereafter, we focus on flow and flow shear effects at the *q* = 2 surface on kinetic RWM stability. To this end, we use “artificial” rotation frequency profile by invoking an error function. We varied Ω at *q* = 2 as Ω = 0~50 krad/s, and for each Ω, we varied the rotation shear *d*Ω/*dψ*_*n*_ at *q* = 2 as *d*Ω/*dψ*_*n*_ = 100~400 krad/s where *ψ*_*n*_ is the normalized magnetic flux. Typical rotation frequency profiles are shown in Fig. 3, which indicates that the flow shear is changed with keeping the flow amplitude at the *q* = 2 surface. Note that recently, the flow and flow shear effects on RWM stability were investigated in ref. 21. This study invokes the self-consistent approach, which considers the effect of particle kinetics of modifying the MHD modes, and analysed the stability with small flow. On the other hand, in this study, we consider the effect of fast flow on the energy exchange term, *δW*_*K*_.

**Figure 4:**
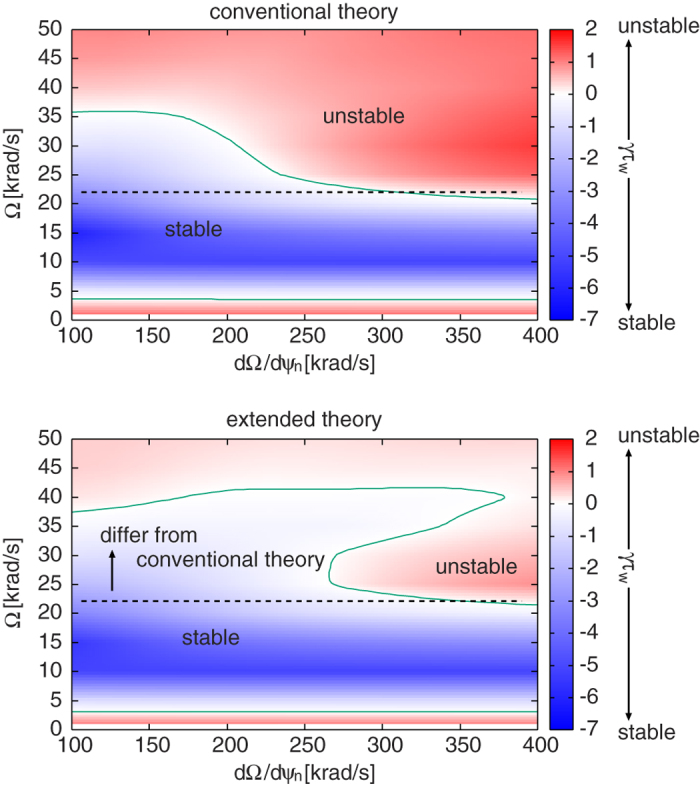
Comparison of RWM growth rates in the flow shear-flow diagram for the conventional and extended kinetic MHD theories.The extended theory indicates the enlarged stable regions and reduced RWM growth rates in the unstable region.

Performing a scan using the rotation frequency profiles, we can obtain the stability diagram in a “flow shear”-“flow” plane as shown in Fig. 4. The RWM growth rate is computed by equation (2). Here we should make a remark that using the dispersion relation (2) in the present formalism has a subtle problem. It is because the dispersion relation assumes the negligible plasma inertia^10^, which is not the case for fast plasma flow. The synergetic effect of plasma inertia and extended *δW*_*K*_ terms will be studied in future, and in this paper we exclusively focus on the *δW*_*K*_ effects. In Fig. 4, we find a narrow band of rotation frequency amplitude (between 0 and 5 krad/s), where the RWM is unstable. This would be attributed to the drift reversal effect of the precession frequency, which occurs in a wide range of radial location for the equilibrium studied in this study. The stabilization effect in the rotation frequency range 5 krad/s ≤ Ω ≤ 35 krad/s stems from the resonance with the precession frequency of passing ions (~10 krad/s), the precession frequency of trapped ions (~10 krad/s), and the bounce frequency of trapped ions (~20~30 krad/s) [see the numerator of Eq. (6)]. Finally, comparing two diagrams in Fig. 4, we find that the extended kinetic MHD theory indicates the enlarged stable region, and reduced RWM growth rates in the unstable region. This fact shows the importance of the modified *δW*_*K*0_ and the additional kinetic contributions, *δW*_*K*1_, *δW*_*K*2_, and *δW*_*K*3_. Unstable regions in both theories stem from the smallness of the damping effect by the resonance with the particles’ drift frequencies. However, in the extended theory, the smallness of the damping effect is compensated.

## Discussion

**Figure 5:**
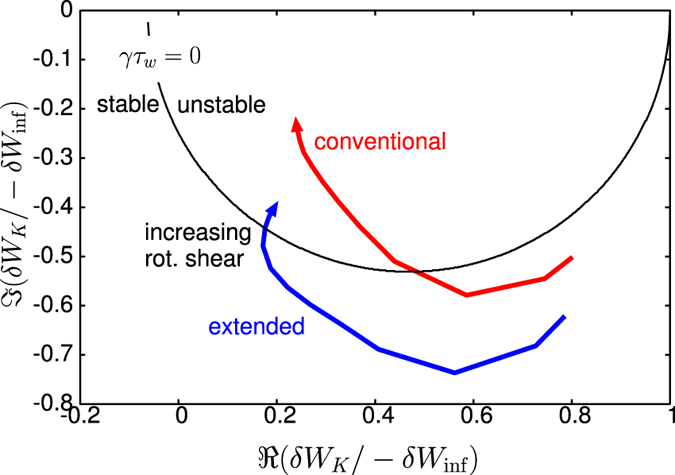
Trajectory of *δW*_*K*_ in the ℜ*δW*_*K*_ - ℑ*δW*_*K*_ plane for conventional and extended theories with Ω = 35(krad/s) with indication of marginal stability.The arrow indicates the increasing of the flow shear.

**Figure 6:**
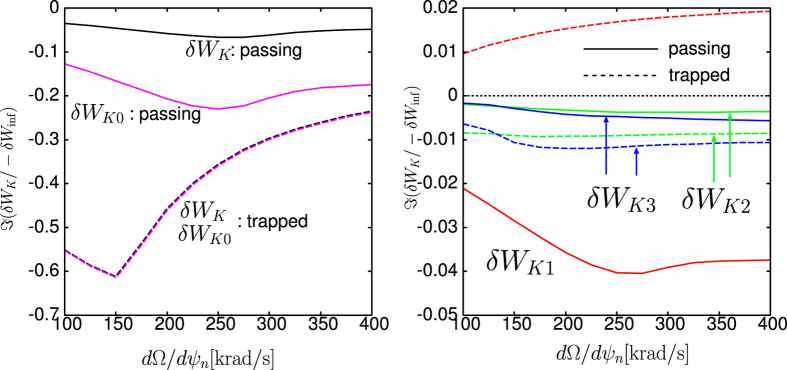
(Left) Imaginary parts of original *δW*_*K*_ and modified *δW*_*K*0_ as functions of flow shear for Ω = 35(krad/s).(Right) Imaginary parts of the additional *δW*_*K*_’s as functions of rotation shear for Ω = 35(krad/s). The new terms are due to the Coriolis (*δW*_*K*1_) and centrifugal (*δW*_*K*2_) acceleration, and the modification to particle distribution function (*δW*_*K*3_).

To explain the observation in the numerical Results, we focus on the case with rotation frequency amplitude Ω = 35(krad/s). The stabilising effect of the new channels of energy exchange, *δW*_*K*_ terms, can be clearly shown as follows. Since the RWM stability is determined by the dispersion relation (2) and *δW*_*b*_ and *δW*_∞_ are given, real constants, the RWM stability can be evaluated in the ℜ*δW*_*K*_ − ℑ*δW*_*K*_ plane. Figure 5 shows the trajectories of *δW*_*K*_ of the conventional and extended theories in the ℜ*δW*_*K*_ − ℑ*δW*_*K*_ plane for Ω = 35(krad/s) with indication of marginal stability. The difference of the trajectory between the conventional and extended theories is attributed to the modified *δW*_*K*0_ term and the additional *δW*_*K*_ terms. Figure 5 indicates that the modified and new terms are working as stabilising, since they shift the trajectory to more stable region. This effect reflects the extended stable region shown in Fig. 4. To see which *δW*_*K*_ term is significant, we plot the imaginary parts of each *δW*_*K*_ for Ω = 35(krad/s) as functions of *d*Ω/*dψ*_*n*_ in Fig. 6. We have picked up the contributions from passing and trapped ions since the electrons’ effect can be negligible in the present calculation. As shown in Fig. 6, the most significant stabilising effect is the modification of *δW*_*K*0_ term for passing ions and the secondary contribution stems from the Coriolis acceleration of passing and trapped ions. The difference in *δW*_*K*_ and *δW*_*K*0_ is attributed to the modification of the precession frequency by the Coriolis and centrifugal acceleration. Since for trapped particles, the modification by the Coriolis acceleration vanishes due to the dependence on *σ*, the sign of the particle parallel velocity, the difference is significant in passing particles. In contrast, as for the centrifugal acceleration and rotation shear effect, both trapped and passing ions are not negligible, however their contributions are small. Even though the explicit rotation shear effect of *δW*_*K*3_ is small, changing rotation shear at a constant *q* = 2 rotation does affect stability when the rotation level is near marginal and this effect is modified by the extended theory presented here.

Summarizing, we have generalised the kinetic-MHD theory to include flow effect self-consistently. The generalisation is realised by introducing the guiding centre Lagrangian with flow and modified particle distribution function. As a result, the energy change induced by kinetic resonance, *δW*_*K*_, which plays a significant role in the kinetic-MHD theory, is generalised to constitute of four terms [see Eq. (8)]. The original *δW*_*K*_ is generalised due to the modification of particles’ drift frequencies yielding *δW*_*K*0_, which has different resonance condition. The extended theory has additional *δW*_*K*_’s related to the Coriolis and centrifugal acceleration and flow shear (modification to particle distribution function). These are newly found channels for energy exchange between macroscopic (MHD-scale) motion and microscopic (particle-scale) dynamics, which are induced by macroscopic plasma flow. A module to compute the conventional and additional *δW*_*K*_’s is implemented in a linear MHD code, MINERVA/RWMaC. We examine the flow and flow shear effects on the RWM stability based on the extended theory. We have invoked a numerical MHD equilibrium and varied the flow and flow shear. It is found that the extended theory exhibits the enlarged stable region compared with the conventional theory, and that the RWM growth rates are reduced in the extended theory. We found that the *δW*_*K*0_ modified due to the change of particles’ drift frequencies stabilises the RWM and the new *δW*_*K*_ term related to the Coriolis acceleration plays a secondary role for stabilisation while the effects related to the centrifugal acceleration and rotation shear are relatively small. Finally, we would like to comment on one missing but important physics in the present theory. We have omitted the effects of energetic particles in this study. The existence of the energetic particles can influence the present calculation by introducing the additional *δW*_*K*_ term. This effect can be treated in the present theoretical framework and presented in the near future. This extension will make it possible to investigate the effect of rotation frequency profile on the RWM stability quantitatively and to compare the present theory with experiments.

## Methods

The derivation of equation (5) is described in ref. 15, and the detail of the MINERVA/RWMaC code is described in ref. 19.

## Additional Information

**How to cite this article**: Shiraishi, J. *et al*. Flow-Induced New Channels of Energy Exchange in Multi-Scale Plasma Dynamics – Revisiting Perturbative Hybrid Kinetic-MHD Theory. *Sci. Rep.* **6**, 25644; doi: 10.1038/srep25644 (2016).
